# Strengthening multisectoral coordination on antimicrobial resistance: a landscape analysis of efforts in 11 countries

**DOI:** 10.1186/s40545-021-00309-8

**Published:** 2021-02-28

**Authors:** Mohan P. Joshi, Tamara Hafner, Gloria Twesigye, Antoine Ndiaye, Reuben Kiggundu, Negussu Mekonnen, Ndinda Kusu, Safoura Berthé, Edgar Peter Lusaya, Alphonse Acho, Robert Tuala Tuala, Ayasha Siddiqua, Henri Kaboré, Soukeyna Sadiya Aidara, Javier Guzman

**Affiliations:** 1 USAID Medicines, Technologies, and Pharmaceutical Services (MTaPS) Program, Management Sciences for Health, Arlington, VA USA; 2 USAID Medicines, Technologies, and Pharmaceutical Services (MTaPS) Program, Management Sciences for Health, Abidjan, Côte d’Ivoire; 3 USAID Medicines, Technologies, and Pharmaceutical Services (MTaPS) Program, Management Sciences for Health, Kampala, Uganda; 4 USAID Medicines, Technologies, and Pharmaceutical Services (MTaPS) Program, Management Sciences for Health, Addis Ababa, Ethiopia; 5 USAID Medicines, Technologies, and Pharmaceutical Services (MTaPS) Program, Management Sciences for Health, Nairobi, Kenya; 6 USAID Medicines, Technologies, and Pharmaceutical Services (MTaPS) Program, Management Sciences for Health, Bamako, Mali; 7 USAID Medicines, Technologies, and Pharmaceutical Services (MTaPS) Program, Management Sciences for Health, Dar es Salaam, Tanzania; 8 USAID Medicines, Technologies, and Pharmaceutical Services (MTaPS) Program, Management Sciences for Health, Yaoundé, Cameroon; 9 USAID Medicines, Technologies, and Pharmaceutical Services (MTaPS) Program, Management Sciences for Health, Kinshasa, Democratic Republic of the Congo; 10 USAID Medicines, Technologies, and Pharmaceutical Services (MTaPS) Program, Management Sciences for Health, Dhaka, Bangladesh; 11 USAID Medicines, Technologies, and Pharmaceutical Services (MTaPS) Program, Management Sciences for Health, Ouagadougou, Burkina Faso; 12 USAID Medicines, Technologies, and Pharmaceutical Services (MTaPS) Program, Management Sciences for Health, Dakar, Senegal

**Keywords:** Antimicrobial resistance, Global Health Security Agenda, International Health Regulations, Joint external evaluation, Multisectoral coordination, One Health

## Abstract

**Background:**

Increasingly, there has been recognition that siloed approaches focusing mainly on human health are ineffective for global antimicrobial resistance (AMR) containment efforts. The inherent complexities of AMR containment warrant a coordinated multisectoral approach. However, how to institutionalize a country’s multisectoral coordination across sectors and between departments used to working in silos is an ongoing challenge. This paper describes the technical approach used by a donor-funded program to strengthen multisectoral coordination on AMR in 11 countries as part of their efforts to advance the objectives of the Global Health Security Agenda and discusses some of the challenges and lessons learned.

**Methods:**

The program conducted a rapid situational analysis of the Global Health Security Agenda and AMR landscape in each country and worked with the governments to identify the gaps, priorities, and potential activities in multisectoral coordination on AMR. Using the World Health Organization (WHO) Joint External Evaluation tool and the WHO Benchmarks for International Health Regulations (2005) Capacities as principal guidance, we worked with countries to achieve key milestones in enhancing effective multisectoral coordination on AMR.

**Results:**

The program’s interventions led to the achievement of key benchmarks recommended actions, including the finalization of national action plans on AMR and tools to guide their implementation; strengthening the leadership, governance, and oversight capabilities of multisectoral governance structures; establishing and improving the functions of technical working groups on infection prevention and control and antimicrobial stewardship; and coordinating AMR activities within and across sectors.

**Conclusion:**

A lot of learning still needs to be done to identify best practices for building mutual trust and adequately balancing the priorities of individual ministries with cross-cutting issues. Nevertheless, this paper provides some practical ideas for countries and implementing partners seeking to improve multisectoral coordination on AMR. It also demonstrates that the WHO benchmark actions, although not intended as an exhaustive list of recommendations, provide adequate guidance for increasing countries’ capacity for effective multisectoral coordination on AMR in a standardized manner.

## Background

Antimicrobial resistance (AMR) is internationally recognized as a global threat to humans, animals, the environment, and consequently the global economy and health security. It is estimated that 700,000 people die annually from AMR, a rate that is projected to increase to 10 million by 2050 if adequate and decisive action is not taken [1]. Globally, the World Bank estimates that 24 million people could fall into extreme poverty by 2030 because of AMR and cumulative economic costs of AMR could be up to $120 trillion by 2050, if the problem is left unchecked [2]. AMR threatens progress on many of the Sustainable Development Goals (SDGs), the highest potential impact being on SDG 3, which focuses on health and well-being [2]. AMR also adversely affects the eradication of poverty and hunger (SDG 1), achieving food security and sustainable agriculture (SDG 2), availability of safe water and sanitation (SDG 6), boosting economic growth (SDG 8), and reducing inequality (SDG 10) [3, 4]. AMR threatens the availability of effective antimicrobials to treat infectious diseases, making AMR containment a critical concern for both global health and the global economy [2]. Universal health coverage (SDG 3.8) has the potential to be a key driver in the solution to overcoming AMR through expanded service coverage, increased quality and safety in service provision, improved financing, greater availability of quality data, and enhanced regulation and governance [2]. However, expanding access to medicines without adequate attention to their quality and use could result in a rise of both inappropriate use and availability of substandard and falsified medicines [5, 6].

Previous AMR containment efforts were often siloed, focusing mainly on the human health sector. However, that approach is no longer considered adequate due to potential implications of widespread antimicrobial use, not just in human health, but also in animal health; the agricultural, husbandry, and fishery sectors; as well as environmental contamination through antimicrobial residual discharge. It is now recognized that food from animals can transmit resistant infections to humans [7]. Hence, focusing containment effects primarily in one sector limits the potential for comprehensive and sustained improvements. There is now general acceptance that the inherent complexities of AMR containment require a coordinated multisectoral effort grounded in the One Health approach [8–10]. One Health is a tripartite initiative involving the World Health Organization (WHO), Food and Agriculture Organization (FAO), and the World Organisation for Animal Health (OIE) aimed at multisectoral coordination (MSC) to address public health threats at the interface of humans, animals, and the environment. MSC is the systemic engagement and deliberate coordination of different stakeholder groups—such as the health, agriculture, environment, trade, and education sectors, civil society, and private sector groups—to jointly achieve a goal [4, 9]. The nature of MSC is therefore not restricted to the interface of human, animal, and environmental health but also includes working across and within stakeholder groups and sectors in each health domain (e.g., involvement of trade, education, and private sectors within human or environmental health).

Several global activities have occurred over the past two decades that have helped advance MSC. In May 2005, the World Health Assembly adopted the International Health Regulations (IHR) (2005), which aim to prevent, protect against, control, and respond to public health threats and their underlying causes. IHR (2005) has 19 technical areas, of which AMR is one. The World Health Assembly also adopted the global action plan on AMR based on the One Health approach in 2015 [7]. Member states have been urged to develop their own national action plan on AMR (NAP-AMR) to align with the five strategic objectives of the global action plan [7]. As of January 2021, 143 countries had a NAP-AMR and 43 countries were developing theirs [11]. Countries can use the WHO’s Joint External Evaluation (JEE) tool, launched in 2016, to measure progress on their IHR capacity [12]. The second version of the JEE tool, released in 2018, includes indicator P.3.1, “Effective multisectoral coordination on AMR” [13]. This indicator was absent in the 2016 edition of the tool. WHO has released the benchmarks for IHR Capacities to be used along with the JEE tool to help countries define actionable steps for reaching the defined capacity levels in the various technical areas, including AMR [14]. The JEE and WHO benchmarks categorize countries into five levels based on their capacity (Table 1).

**Table 1:** Actions required by JEE 2 and the WHO benchmarks for IHR capacities to achieve the various capacity levels for indicator P.3.1: effective multisectoral coordination on AMR

Capacity score	JEE 2 [13]	WHO benchmarks for IHR capacities [14]
No capacity—1	No national action plan on AMR (NAP-AMR)	No NAP-AMR
Limited capacity—2	NAP-AMR under development or involves only one sector/ministry MSC mechanism established with government leadership	Establish a national multisectoral AMR coordinating committee Undertake a situation analysis to identify major risks for development and transmission of AMR and where the impact of resistance would be greatest Identify programs and activities relating to key AMR objectives that need to be developed or scaled up Identify a health ministry lead for AMR, develop clear terms of reference. and coordinate activities of the relevant ministries on AMR and stewardship
Developed capacity—3	NAP-AMR developed; addresses at least human and animal sectors MSC coordination functional with regular meetings	Develop a plan of action to address AMR in line with the Global Action Plan (GAP) on AMR Submit a plan for approval through relevant governance mechanisms (such as office of head of state, cabinet, or ministries of health and agriculture) Develop terms of reference for a multisectoral governance mechanism with clear lines of accountability between the AMR coordinating committee and the high-level One Health group Organize effective coordination through regular meetings
Demonstrated capacity—4	Multisectoral NAP-AMR approved; in line with GAP; operational plan and monitoring arrangement in place	Identify priority actions (based on risk and feasibility) from the NAP, develop an implementation plan with responsible agencies with established timelines, and begin implementation of these actions Develop and implement a NAP-AMR monitoring framework Review plans and progress through regular meetings of the AMR governance committee Identify and map sustained funding for planned activities in the AMR national action plan
Sustainable capacity—5	Multisectoral NAP-AMR has identified funding sources; being implemented; monitoring in place	Sustain funding for planned activities in the NAP-AMR Ensure key activities are incorporated in plans and budgets of relevant programs and agencies Ensure regular monitoring of progress with data submitted to regional and global levels Define clearly specified actions within planning and governance mechanisms for all key sectors involved Identify potential barriers and/or challenges to implementing the NAP and approaches to overcome these barriers

The Global Health Security Agenda (GHSA), established in 2014, brings together stakeholders, including countries, international organizations, and the private sector, to accelerate progress on attaining security from global health threats posed by infectious diseases. GHSA is an expanding partnership, supporting 69 countries in improving their capacity in 19 technical areas, including AMR, and uses the JEE to measure countries’ progress in these areas [15]. The GHSA’s AMR Action Package emphasizes multisectoral engagement and collaboration as a pivotal approach to combating AMR [16].

Since its inception in September 2018, the US Agency for International Development (USAID) Medicines, Technologies, and Pharmaceutical Services (MTaPS) program has been a key mechanism for USAID’s GHSA support to partner countries in the areas of antimicrobial stewardship (AMS), infection prevention and control (IPC), and MSC on AMR (MSC-AMR). One of MTaPS’ aims is to help its GHSA-supported countries progress toward the next level of JEE capacity for effective MSC-AMR (Table 1). The program supports MSC-AMR in 11 countries—Bangladesh, Burkina Faso, Cameroon, Côte d’Ivoire, Democratic Republic of Congo (DRC), Ethiopia, Kenya, Mali, Senegal, Tanzania, and Uganda. (The program in Ethiopia ended in December 2020.) This paper describes the program’s technical approach and key implementation milestones and discusses some of the challenges and lessons learned.

## Methods

The program’s mandate on AMS, IPC, and MSC-AMR focuses on human health. However, the scope of MSC-AMR inherently spans multiple sectors and supports One Health principles. The program’s technical implementation approach focuses on strengthening leadership, policy, governance, and enabling environment; building capacity; and supporting monitoring and feedback and self-learning in the three mandated technical areas. The JEE tool and the WHO benchmarks are the principal guidance documents for MTaPS’ GHSA/AMR technical approach. The program also relies on other guidance documents and tools from the WHO/FAO/OIE tripartite.

In preparation for implementation, MTaPS first conducted a desk review, followed by a scoping visit to each country. The desk review included key GHSA and AMR country documents, including JEE baseline reports. We conducted scoping visits in ten countries between October 2018 and March 2019. The scoping visit for Bangladesh was in December 2019. The purpose of the scoping visits was to conduct a rapid situational analysis to better understand the AMR landscape with respect to key stakeholders on ongoing activities, identify strengths, gaps, and priorities in the three mandated areas, including MSC, and triangulate information gathered from the desk review. Stakeholders typically included representatives from the ministry of health, national medicines regulatory authority, USAID/Mission, United States Centers for Disease Control and Prevention (CDC), WHO, FAO, schools of medicine and pharmacy, hospitals, professional associations (medical, pharmacy, and nursing), and the animal sector. The visits enabled us to build relationships with country stakeholders and increase attention to AMR, including from a GHSA perspective. Based on the rapid situational analysis, we worked in concert with governments to identify potential activities in MSC-AMR and develop work plans.

## Results

The desk reviews and scoping visits helped identify strengths, gaps, and priorities for MSC-AMR, and informed the program’s interventions, which included specific benchmark actions and other activities that contributed to the achievement of the benchmarks.

### Identify strengths, gaps, and priorities for MSC-AMR

Although the version of the JEE tool used for the baseline evaluations did not include an indicator for MSC-AMR, the reports outlined some of the challenges and recommended actions pertinent to MSC. The primary issues identified in the JEE baseline reports included: the need to finalize, validate, or disseminate NAPs-AMR, establish multisectoral technical groups to implement AMR plans, and strengthen the capacity of facilities and entities that would play roles in One Health policies (Table 2). Another common theme was the need to increase communication and awareness about AMR in the animal, agricultural, food, and environmental sectors.

**Table 2:** Selected recommendations relevant to MSC-AMR from the JEE baseline reports

Country	Date of JEE baseline	Recommendations for priority actions
Bangladesh	May 2016	Finalize the National Action Plan on AMR in a manner that is aligned with the Global Action Plan. Public health and animal sectors to develop further collaborative projects focusing on surveillance of AMR and antimicrobial use [17]
Burkina Faso	Dec 2017	Validate and implement the National Multisectoral Action Plan to fight against antimicrobial resistance. Establish a coordination and collaboration body for the different sectors involved in the fight against antimicrobial resistance, responsible among other things for the development and implementation of standard operating procedures [18]
Cameroon	Sep 2017	Finalize, validate, and disseminate the national plan for the detection and reporting of antimicrobial resistant pathogens by integrating the private sector. Develop, validate, and disseminate a national plan for nosocomial infection control programs. Establish a multidisciplinary and multisectoral technical group to implement this plan according to the One Health approach [19]
Côte d'Ivoire	Dec 2016	Develop a national institutional framework for the prevention and control of infections and AMR and establish set roles and responsibilities at all levels of the health pyramid in human and animal medicine. Develop a strategic action plan, based on WHO technical recommendations that is adapted to the reality on the ground and accompanied by a budgeted operational plan. Strengthen the capacity of all facilities with an important role to play under the new One Health policy. Increase advocacy and awareness-raising about AMR in the animal, agricultural, food and environmental sectors [20]
DRC	Mar 2018	Design and implement a multisectoral national action plan for the detection and reporting of antimicrobial resistant pathogens, and for antimicrobial stewardship. Strengthen the capacity of staff and structures in the fight against antimicrobial resistance [21]
Ethiopia	Mar 2016	Ensure intersectoral collaboration and continuous stakeholder communication and behavioral change within animal health and public health sectors. Implement an antimicrobial resistance stewardship program within animal health and public health sectors [22]
Kenya	Feb 2017	Strengthen and fully implement antimicrobial stewardship activities in the human and animal health sectors [23]
Mali	Jun 2017	Develop and implement a comprehensive national plan for the detection and notification of priority antimicrobial resistant pathogens, covering human and animal health, agriculture, food and the environment as part of the One Health approach. Develop an information and awareness program on AMR in the different sectors [24]
Senegal	Nov 2016	Draw up a national action plan to tackle antimicrobial resistance, considering the global action plan on antimicrobial resistance, which spans both animal and human health. Set up a coordination mechanism for different multisectoral operations concerning antimicrobial resistance. Better coordinate multisectoral operations and strengthen ties between human and animal health laboratories. Strengthen stakeholders’ capacities to implement the national action plan [25]
Tanzania—Mainland	Feb 2016	Develop a national action plan to address antimicrobial resistance. This should align with the Global Action Plan on Antimicrobial Resistance, incorporating action by all relevant sectors, particularly from health, veterinary and agriculture sectors. The first step would be for the government to nominate a national task force and convene a multisectoral group with high-level leadership [26]
Tanzania—Zanzibar	Apr 2017	Develop and implement the multisectoral national action plan on AMR [27]
Uganda	June 2017	Develop a clear implementation plan for the National AMR Action Plan with monitoring and evaluation indicators and clear timelines for human, animal, food, plant, and environmental health sectors [28]

By the time of the scoping visits, some of the issues identified in the JEE baseline had already been addressed. For example, the JEE reports indicated that Bangladesh, Burkina Faso, Cameroon, Côte d’Ivoire, DRC, Mali, Senegal, and Tanzania needed to develop or finalize their NAPs-AMR. However, Bangladesh, Cameroon, Senegal, and Tanzania had already finalized theirs at the time of the scoping visits while the other countries had drafts at various stages of development.

The situational analyses revealed that all countries had an established One Health platform and high-level political commitment to AMR containment. However, some countries had issues with inadequate institutionalization of the platform. Generally, the animal sector was less active on AMR relative to the human sector, and the environmental sector was the least active. All countries were struggling to varying degrees with finalizing or operationalizing their NAPs-AMR and MSC-AMR bodies. The coordination and relationships between the MSC-AMR body and the One Health body were generally unclear and weak. Additionally, there was often no clear synergy observed between the mainstream AMR work and the GHSA’s support in this area. The MSC-AMR bodies were primarily established at the central level, but Bangladesh and Kenya had at least articulated aims to expand multisectoral coordination mechanisms to subnational levels in their national action plan or strategy on AMR. Weaknesses in MSC-AMR were often due to inadequate political support, authority to act, administrative and financial support, AMR data availability or use, and practical know-how on the process and parameters of how such bodies should operate. The MSC-AMR bodies in some cases were established without clear supporting policies regarding funding and human resources. The gaps in capacity and function of the MSC-AMR bodies were constraining the countries’ progress in implementing the NAP-AMR objectives—including those on IPC and AMS—and hence overall progress on AMR containment.

Given the findings from the scoping visits, MTaPS prioritized strengthening the function and technical capacity of multisectoral governance structures as a critical intervention to operationalize NAPs-AMR and strengthen capacity for AMR containment in all its 11 GHSA/AMR-supported countries. We chose the interventions based on the specific guidance on MSC-AMR (indicator P.3.1) now included in the revised 2018 edition of the JEE tool and the corresponding WHO benchmarks recommended actions (hereafter benchmark actions) (Table 1). The program’s interventions included specific benchmark actions and other activities that contributed to the achievement of the benchmarks—finalizing and operationalizing NAPs-AMR; strengthening MSC-AMR governance bodies’ leadership and oversight capabilities, supported by established or revitalized IPC and AMS technical working groups (TWGs); and coordinating AMR activities within and across sectors.

### Help finalize and operationalize NAP-AMR

The program’s support on MSC resulted in three countries finalizing their NAP-AMR, four developing tools to guide implementation, two seeking appropriate funding, and four working to increase communication and awareness of AMR in partial fulfillment of their NAP-AMR objectives (Table 3).

**Table 3:** MTaPS-supported activities to finalize and operationalize NAPs-AMR, in collaboration with MSC-AMR bodies

JEE capacity level	MTaPS-supported activities	Bangladesh	Burkina Faso	Cameroon	Côte d'Ivoire	DRC	Ethiopia	Kenya	Mali	Senegal	Tanzania	Uganda	Total (%) *n* = 11
3	Finalize/submit a NAP-AMR		✔		✔				✔				3 (27%)
4	Develop a NAP-AMR operational plan			✔	✔								2 (18%)
4	Develop a NAP-AMR monitoring framework	✔						✔					2 (18%)
4	Identify sustained funding for NAP-AMR activities						✔			✔			2 (18%)
–	Bolster AMR communication (NAP-AMR activity)	✔						✔			✔	✔	4 (36%)

Developing a NAP-AMR and submitting the plan for approval through relevant governance mechanisms is a key benchmark action for developed capacity (level 3) (Table 1). We worked with Burkina Faso, Côte d’Ivoire, and Mali to help finalize their NAP-AMR. In Côte d’Ivoire, we supported the AMR TWG to develop, finalize, and use a national AMR policy and governance manual and an advocacy document to accompany the NAP-AMR. The NAP-AMR has since been approved and is used to advocate for high-level commitment within the health, education, agriculture, and environment ministries. In Mali, MTaPS supported the Multisectoral Coordination Committee for AMR (Groupe de Coordination Multisectorielle National-Résistance aux Antimicrobiens, GCMN-RAM) to revise their NAP-AMR and submit it for inputs from the One Health platform, Ministry of Health (MOH), Ministry of Livestock and Fisheries, Ministry of Environment, and the Ministry of Agriculture.

In some countries, MTaPS supported the development of implementation or operational plans and monitoring and evaluation (M&E) frameworks to guide NAP-AMR implementation. Both steps are recommended benchmark actions to move to demonstrated capacity (level 4). The human, animal, agriculture, and environment sectors and partner organizations in Cameroon collaborated to draft an operational plan for their NAP-AMR. Bangladesh and Kenya developed a multisectoral M&E framework for their NAP-AMR.

National stakeholders in Ethiopia and Senegal drafted and submitted funding concept notes to the Tripartite AMR Multi-Partner Trust Fund (MPTF) with support from MTaPS. MPTF is a newly established vehicle to fund partner countries’ intersectoral collaboration to implement the NAP-AMR. This effort contributes to a level 4 benchmark action—identifying and mapping sustained funding for planned activities in the AMR national action plan (Table 1).

DRC and Ethiopia conducted the Tripartite AMR Country Self-Assessment Survey (TrACSS) for 2019–2020 in collaboration with MTaPS. TrACSS is a component of the global framework to monitor NAP-AMR implementation status, and countries report the self-assessment results to the tripartite group for global summary. Conducting this annual self-assessment helps illustrate the country’s progress in building an effective and sustainable collaborative multisectoral work and response to AMR.

Implementation has also included work on communication to increase AMR awareness in Bangladesh, Kenya, Tanzania, and Uganda. Acting on Tanzania’s NAP-AMR, MTaPS led the development of a multisectoral communication strategy to increase awareness and understanding of AMR across all sectors. The country’s Multisectoral Coordination Committee on AMR approved the strategy in February 2020. The government and AMR stakeholders began using the standardized messages from the AMR communication strategy during the World Antimicrobial Awareness Week 2020. Furthermore, the document is used by AMR stakeholders to promote behavior change to improve the appropriate use of antimicrobials among health care providers and the wider community.

Uganda’s NAP-AMR also has the promotion of AMR awareness as one of its objectives. MTaPS helped develop and validate guidelines for infection prevention and appropriate use of antimicrobials in all the leading farming systems in Uganda—cattle, fish, goats and sheep, pigs, and poultry. The Ministry of Agriculture, Animal Industry, and Fisheries leadership plan to use these guidelines widely. We also collaborated with stakeholders in Bangladesh, Burkina Faso, Côte d’Ivoire, Ethiopia, Kenya, Tanzania, and Uganda to organize AMR awareness events during the World Antimicrobial Awareness Week in November 2019 and/or 2020. For example, in Tanzania in 2019 the program brought together 700 participants for an AMR symposium organized by Tanzania Pharmaceutical Students’ Association. In Bangladesh, the program facilitated two round table discussions on antimicrobial use around World Antimicrobial Awareness Week 2020 with participants from the national government, FAO, WHO, Fleming Fund, pharmaceutical industry and trade associations, professional associations, the media, and other implementing partners.

### Strengthen leadership, governance, and oversight capabilities of MSC-AMR bodies

While the name of the MSC-AMR body varies across countries, in all cases it is a mandated governance mechanism that represents the highest level of multisectoral coordination on AMR in the country. The MSC-AMR body typically has TWGs, which are responsible for the technical implementation of the various strategic objectives in the country’s NAP-AMR, such as IPC, AMS, and surveillance. Although all the countries had an MSC-AMR body, their functionality was often weak. We therefore focused on working with these bodies to develop or revise their TORs (including the formation of TWGs) to strengthen governance mechanisms, and organize effective coordination through regular meetings, both of which are recommended level 3 benchmark actions for improving the function of MSC-AMR bodies (Table 1). Additionally, MTaPS helped establish or strengthen the AMR Secretariat in seven countries (Table 4).

**Table 4:** MTaPS-supported activities to strengthen leadership, governance and oversight capabilities of MSC-AMR bodies

JEE capacity level	MTaPS-supported activities	Bangladesh	Burkina Faso	Cameroon	Côte d'Ivoire	DRC	Ethiopia	Kenya	Mali	Senegal	Tanzania	Uganda	Total (%) *n* = 11
2	Establish/revitalize MSC-AMR body: IPC TWG			✔	✔	✔	✔	✔	✔		✔	✔	8 (73%)
	AMS TWG			✔	✔	✔	✔	✔	✔	✔	✔	✔	9 (82%)
	AMR Secretariat		✔		✔		✔	✔	✔	✔	✔		7 (64%)
3	Develop terms of reference for an IPC/AMS TWG or AMR Secretariat		✔	✔	✔	✔	✔	✔	✔	✔	✔	✔	10 (91%)
3	Organize effective coordination through regular meetings	✔	✔	✔	✔	✔	✔	✔	✔	✔	✔	✔	11 (100%)
4	Facilitate regular AMR governance committee meetings (to review NAP-AMR implementation status)	✔			✔	✔	✔	✔		✔			6 (55%)

To help bolster AMR governance in Ethiopia, we supported the Pharmaceutical and Medical Equipment Directorate to conduct an assessment to understand the level of engagement of government stakeholders in AMR containment. The results revealed a weak coordination mechanism for AMR stakeholders; a lack of a monitoring and evaluation framework to measure progress; no central reporting channel on AMR-related activities; and the absence of functional TWGs in line with the One Health approach to combat AMR. As a result, Ethiopia finalized the design of a three-tiered national AMR governance and coordination structure, comprising a National Inter-Ministerial Committee at the top, the National AMR Advisory Committee (NAMRAC), and six multisectoral TWGs. NAMRAC, whose membership includes the human health, animal health, food, and environmental sectors, revised its TOR to align with the WHO’s sample TOR for an MSC-AMR body [29]. The revision amended NAMRAC’s role from an advisory committee to an MSC committee.

Instituting routine, organized meetings for the MSC-AMR bodies has led to their demonstrated leadership of some AMR-related activities. For example, the lack of regular communication among GCMN-RAM members in Mali had been a major hindrance to coordination. With our technical and financial support, GCMN-RAM conducted its first two meetings with participants from the health and social affairs, agriculture, livestock and fisheries, and environment ministries, and international organizations, including USAID, FAO, OIE, and WHO. GCMN-RAM now has a validated TOR and led the revision of the country’s NAP-AMR. MTaPS has since worked with the GCMN-RAM and the Hygiene Sub-Directorate of the Direction Générale de la Santé to revise IPC guidelines for the human sector and draft associated training materials. A subsequent validation workshop in January 2020 had the participation of IPC stakeholders at the central and regional levels, including all the heads of hygiene and sanitation divisions in Mali’s 10 regions. The final guidelines are now available and include training curriculum, participants’ manual, and training modules on IPC topics.

MTaPS facilitated multisectoral meetings of the MSC-AMR bodies in Bangladesh, Côte d’Ivoire, DRC, Ethiopia, Kenya, and Senegal to review the implementation status of their NAPs-AMR. For example, the Pharmaceuticals Unit and the Pharmaceutical and Medical Equipment Directorate of the Ministry of Health in Ethiopia conducted a workshop with regional health bureaus to review implementation status. As a result, each region agreed to assign an AMR focal person to coordinate the region’s actions against AMR, develop a regional action plan, and strengthen or reestablish regional AMR advisory committees. We also supported the AMR secretariat in adapting the national strategy on AMR to a regional action plan in the Somali Regional State. This process included federal and regional offices of human health, animal health, academia, and the regional laboratory, and demonstrated how the larger national strategy can align with regional realities to prevent and contain AMR.

In Uganda, the One Health TWG provides leadership for the National Antimicrobial Resistance Sub-Committee (NAMRsC), the MSC-AMR body. The chair of the One Health TWG rotates every 3 months between the MOH, Ministry of Agriculture, Animal Industry and Fisheries, and the Ministry of Water and Environment. This strengthens leadership, incentivizes ownership and collaboration among the ministries, and helps motivate the NAMRsC members appointed by their respective ministries. The NAMRsC appointed one person each from the IPC technical working committee, AMS technical working committee, and the Ministry of Water and Environment to support the 2019 national IPC survey conducted by the MOH, in collaboration with MTaPS, WHO, and other local implementing partners. The team has since supported surveys in AMS areas, including surveillance of the consumption of antibiotics at various health facilities. These survey findings have been shared in the quarterly NAMRsC meetings and used by the MOH to identify priority activities to improve the national IPC program. NAMRsC also collaborates with other programs, such as the Fleming Fund, to implement AMR-related activities throughout the country. These examples demonstrate how such MSC-AMR bodies have moved beyond meetings to specific actions to support AMR containment.

### Build AMR, IPC, and AMS technical capacity of MSC bodies

MTaPS worked with the MSC-AMR bodies in nine countries to establish or revitalize multisectoral or multidisciplinary IPC or AMS TWGs (Table 4). In some cases, this meant revising the TORs of the MSC-AMR body to include the newly established TWGs. MTaPS support to the MSC-AMR bodies and their TWGs has contributed to a variety of actions, such as the development of AMS and IPC policies and guidelines; assessments and action plans; trainings materials, including eLearning modules; and training courses, including train-the-trainers (Table 5).

**Table 5:** MTaPS-supported activities to build MSC bodies’ technical capacity in AMR, IPC, and AMS

MTaPS-supported activities	Bangladesh	Burkina Faso	Cameroon	Côte d'Ivoire	DRC	Ethiopia	Kenya	Mali	Senegal	Tanzania	Uganda	Total (%) *n* = 11
Policies, guidelines, strategies, plans on IPC, AMS, and other AMR areas	✔	✔	✔	✔		✔	✔	✔	✔	✔	✔	10 (91%)
Capacity building of members of MSC-AMR bodies				✔		✔	✔	✔		✔		5 (45%)
Capacity building of health care workers and other stakeholders	✔		✔	✔	✔	✔	✔	✔	✔	✔	✔	10 (91%)
Conduct IPC assessment	✔		✔	✔		✔	✔	✔	✔	✔	✔	9 (82%)
Develop IPC action plan	✔			✔			✔		✔	✔	✔	6 (54%)
Conduct AMS assessment		✔	✔	✔	✔		✔	✔	✔	✔		8 (73%)
Develop AMS action plan				✔			✔	✔	✔	✔	✔	6 (55%)
Support IPC working groups in the COVID-19 pandemic response	✔		✔	✔		✔	✔	✔	✔	✔	✔	9 (82%)

In Cameroon, Côte d’Ivoire, DRC, Tanzania, and Uganda, where some TWGs did not exist, we worked with the MSC-AMR body to help establish them (Table 4). For example, Cameroon’s AMR Technical Secretariat and stakeholders from the One Health platform worked with MTaPS to establish IPC and AMS TWGs in accordance with the country’s NAP-AMR. Côte d’Ivoire created multisectoral technical committees for sanitation and IPC, and AMS and sale of illegal drugs with our support. We collaborated with them to develop their TORs, and IPC and AMS roadmaps with prioritized activities to facilitate NAP-AMR implementation. The committee for sanitation and IPC has since led site visits to establish hygiene and IPC committees and evaluate the functionality of existing committees in three health facilities and one veterinary clinic. MTaPS worked with the committee for AMS and sale of illegal drugs to assess the functionality of drug and therapeutics committees in two teaching hospitals and started developing an AMS training curriculum for committee members. In Uganda, the AMS technical working committee was involved in developing a veterinary essential medicines list, which is now finalized with approval from the Ministry of Agriculture, Animal Industry and Fisheries.

In countries where the TWGs already existed, our efforts focused on developing or revising TORs, helping to coordinate regular meetings, and developing policies and guidelines for IPC and AMS (Table 4). In Tanzania, MTaPS supported and participated in the first IPC TWG meeting, where we advised on the TOR contents and assisted the Multisectoral Coordination Committee to develop key IPC indicators and a strategy to oversee and monitor IPC implementation. The IPC TWG has since been coordinating supportive supervision visits to hospitals and the revision of associated guidelines and tools. Kenya’s National Antimicrobial Stewardship Interagency Committee worked with us to review TORs for its four TWGs—advocacy and awareness, research and surveillance, IPC, and AMS—to align them with the NAP-AMR and develop 1-year action plans. Further, our Kenya program engaged county health leadership and management teams in Nyeri and Kisumu counties to advocate for the establishment of One Health governance structures and systems to strengthen IPC and AMS at the county, sub-county, and health facility levels. Nyeri has since established a County Antimicrobial Stewardship Interagency Committee, which is a county-level MSC-AMR structure. In collaboration with partners including FAO, the USAID-funded Infectious Disease Detection and Surveillance program and MTaPS, the committee has developed and launched its 2020–2022 workplan. MTaPS is also helping establish or strengthen existing but weak IPC committees and medicines and therapeutics committees at the county and facility levels in Nyeri and Kisumu to drive IPC and AMS agendas. So, in the case of Kenya, work on governance structures to facilitate MSC-AMR is being cascaded from the national to the county levels, helping to facilitate vertical coordination in the health system.

The functionality of existing multisectoral coordination bodies is perhaps most evident and timely in responding to the COVID-19 pandemic. IPC TWGs or some of their members contributed to national pandemic response in Bangladesh, Cameroon, Côte d’Ivoire, Ethiopia, Kenya, Mali, Senegal, Tanzania, and Uganda (Table 5). In those countries, the program had supported developing AMR, IPC, and AMS policies, guidelines, standard operating procedures, and training modules, some of which IPC TWGs used to adapt to the COVID-19 context to build capacity for the pandemic response. Except for Burkina Faso and DRC, MTaPS collaborated with COVID-19 task forces and MSC-AMR bodies to convert IPC training materials to competency-based training packages for master trainers, who then trained first-line workers on IPC for COVID-19. This showed the broader systemic value and usefulness of MSC-AMR bodies and their TWGs beyond their primary aim of containing AMR.

### Facilitate coordination of AMR activities within and across sectors

Table 6 summarizes the different stakeholder groups MTaPS has collaborated with to advance AMR-related actions across the 11 countries. As outlined in the preceding sections, MTaPS’ technical support has been instrumental in improving engagement and coordination of stakeholders from various sectors in 11 countries, which contributes to the level 3 benchmark action—organize effective coordination through regular meetings (Table 1). Examples of such work include Bangladesh, where a multisectoral meeting in December 2019 had good representation from the human and animal sectors. The meeting resulted in recommendations for strengthening multisectoral approaches for AMR containment, including the formal assignment of sector focal points by a government order, regular meetings of the National Steering Committee and National Technical Committee on AMR, and a publicly available web-based platform to share information and resources related to AMR containment. By January 2021, three more multisectoral meetings had been held. In addition to participants from the human, agriculture, and fisheries sectors, the meetings involved representatives from academia, WHO, FAO, and implementing partners, and in some cases representatives from the environmental sector and professional associations.

**Table 6:** Key stakeholder groups with whom MTaPS has collaborated in country to support actions related to multisectoral coordination on AMR

Country	Government ministries			Academia/research institutions	Professional associations	Civil society organization	International agencies			
	Health	Agriculture/Fisheries/Husbandry	Environment				WHO	FAO	OIE	Other (i.e., international development agencies, implementing partners, nongovernmental organizations)
Bangladesh	✔	✔		✔	✔		✔	✔		✔
Burkina Faso	✔	✔	✔				✔	✔		✔
Cameroon	✔	✔	✔				✔	✔		✔
Côte d’Ivoire	✔	✔	✔	✔	✔	✔	✔	✔	✔	✔
DRC	✔	✔	✔	✔			✔	✔		✔
Ethiopia	✔	✔	✔	✔	✔	✔	✔	✔		✔
Kenya	✔	✔	✔	✔	✔	✔	✔	✔	✔	✔
Mali	✔	✔	✔	✔	✔	✔	✔	✔	✔	✔
Senegal	✔	✔	✔		✔		✔	✔	✔	✔
Tanzania	✔	✔	✔	✔	✔	✔	✔	✔	✔	✔
Uganda	✔	✔	✔	✔	✔	✔	✔	✔	✔	✔

In Tanzania, MTaPS worked to advance MSC by organizing multiple workshops that brought together stakeholders from the FAO, Ministry of Livestock and Fisheries, and the Tanzania Mainland Ministry of Health. At one of these workshops in September 2019, participants, including WHO, CDC, Sokoine University of Agriculture, the environmental sector, and the National Medicines and Therapeutic Committee, carried out a final review of the draft AMS policy guidelines, which were then finalized by the Multisectoral Coordination Committee in November 2019 and approved in December 2020. Similarly, through our efforts in Uganda, major national and subnational IPC stakeholders, representing MOH, Makerere University, WHO, the One Health platform, Infectious Diseases Institute, and other partners, convened and drafted TORs for the national IPC technical working committee. In Burkina Faso, MTaPS collaborated with the Directorate General of Environmental Protection to provide sensitization to a high-level audience on the importance of antimicrobial stewardship and strengthening of the legal framework on AMR during an environmental conventions workshop organized for parliamentarians. As part of its civil society organization engagement strategy in Ethiopia, MTaPS collaborated with the MOH to provide a 3-day training on AMR to 29 volunteers from the Ethiopian Youth and Women Federations on AMR, 21 (72%) of whom were women. Those female volunteers subsequently conducted educational sessions on the rational use of antimicrobials for 520 members of the Addis Ababa Women Federation.

## Discussion

In its 2019 final report to the Secretary-General of the United Nations, the Interagency Coordination Group (IACG) on Antimicrobial Resistance identified four categories of countries with respect to progress made on NAPs-AMR: (1) no plan or strategy on AMR; (2) preparing a plan or in the process of approving a plan; (3) have a plan but experiencing difficulty in implementation; (4) have a plan or strategy that is being implemented [4]. With the finalization of the NAPs-AMR in Burkina Faso, Côte d’Ivoire, and Mali, all 11 countries we support now fit firmly in the third category. Although MSC has not yet been formally evaluated using the revised 2018 version of the JEE tool, the 11 countries currently appear to have level 3 capacity for effective MSC-AMR [13]. The countries have largely addressed the recommended benchmark actions required for this level, including developing and submitting NAPs-AMR for approval, developing TORs for MSC governance mechanisms, and organizing effective coordination through regular meetings (Tables 3, 4 and 5). Some countries have made further progress towards completing some of the actions required for level 4 capacity.

As found in the scoping visits, some aspects of an enabling environment, such as high-level political will, already existed in the 11 countries. However, both the level of political will and awareness of the AMR issue varied among stakeholders and across sectors. By bringing stakeholders together through the strengthening of MSC-AMR, MTaPS has helped increase both political will and awareness across sectors. In Côte d’Ivoire, for instance, the advocacy document developed by the AMR TWG to accompany the NAP-AMR will be used to advocate for high-level commitment and increase awareness across different ministries. Similarly, communication efforts to boost AMR awareness in Tanzania and Uganda will contribute to a more enabling environment.

Many of the countries MTaPS supports lack adequate funding sources and monitoring processes for NAP-AMR implementation, which, in some cases, was due to insufficient details regarding operational plans, costing, and monitoring and evaluation. This issue was compounded by poor data availability and monitoring, and weak diagnostic and regulatory capacities throughout the health system. Adequate monitoring and feedback mechanisms are critical for tracking progress and aligning targets among collaborators [9]. AMR containment efforts were fragmented with limited engagement of animal, agriculture, and environmental sectors. These limitations are similar to those identified by IACG—awareness and political will, finance, coordination, data and technical capacity, and monitoring are the five key challenges commonly faced by countries in implementing their NAPs-AMR [4].

Ongoing interventions in the 11 countries have directly or indirectly helped to address some of those identified challenges by focusing efforts on strengthening governance mechanisms for MSC-AMR. Operationalizing NAPs-AMR through costed and prioritized operational plans with monitoring and evaluation frameworks and the necessary technical support has been a major MTaPS priority. The focus on developing and revising TORs for these bodies helps to ensure clarity about the roles and responsibilities of members and stipulates the structures and processes for accountability and oversight, which are all critical for these mechanisms’ function. WHO provides a sample TOR, which has been a useful resource to help countries strengthen the function of their MSC bodies [29]. We have endeavored to support MSC-AMR bodies to engage with stakeholders as diverse as national laboratories and international donors to achieve the necessary stakeholder coordination to align and scale up their efforts and maximize impact on AMR containment (Table 6). Convincing ministries to engage is important for boosting the AMR agenda, but implementation also relies on department-level decision-making on necessary budget allocations, whether through finding additional resources or realigning existing programs [30]. In low- and middle-income countries, MSC-AMR bodies may play an even more critical role in helping to prioritize NAP-AMR activities based on what can be achieved with existing programs versus those that require entirely new resources [30].

MTaPS support for both the national MSC-AMR body and its associated IPC/AMS TWGs allowed the program to leverage its activities between the different levels of the system (e.g., national, county, and facility levels) and enhance vertical coordination in countries such as Côte d’Ivoire, Kenya, and Tanzania. Further, TWGs serving specific technical areas can reach downstream to targeted groups that can directly and effectively support technical interventions, as seen in Côte d’Ivoire and Tanzania, where the TWGs supervised and supported facilities to advance IPC and AMS actions.

### Common challenges

The emergence of COVID-19 has created an unprecedented global challenge and since March 2020, MSC-related program activities in most of the countries have slowed due to countries shifting their priorities toward addressing the pandemic. It is important to note, however, that some of the IPC TWGs created or strengthened through our interventions have quickly used their enhanced capacity to support their countries’ pandemic response through, for example, assisting with or advising on the development of training curriculum and the development of national guidelines and action plans.

Whereas the animal sector is becoming more engaged in the AMR response in the 11 countries, environmental sector engagement continues to be weak. Coordination across diverse sectors that normally work in silos is not always easy. The principle of multisectoral collaboration is now widely acknowledged and accepted, however, the complexity of coordination within and across sectors continues to be a challenge [31]. At the operational level, identifying best practices for different contexts, including how to adequately balance the priorities of individual ministries with cross-cutting issues, is greatly needed [32]. MTaPS’ experience has confirmed that formal and mandated multisectoral structures, often involving high-level governmental officials from various sectors, are substantially helpful to address this challenge. The MSC-AMR bodies need to focus on action-oriented approaches. Although joint meetings, commitments, and advocacy are critical initial steps, MSC-AMR bodies need to help catalyze specific and coordinated containment actions by various stakeholders from both the public and private sectors. The bodies need to strategize the design, implementation, documentation, and dissemination of the results of co-activities that demonstrate a One Health spirit. WHO, FAO, and OIE, as part of their tripartite efforts, have provided guidance and support to member states on establishing and sustaining MSC mechanisms to help advance AMR containment. MTaPS’ collaboration with these United Nations bodies has been instrumental in our efforts to support our target countries in strengthening their MSC-AMR bodies (Table 6).

Despite the progress made in improving AMR awareness, critical gaps remain that undermine MSC-AMR efforts and the successful implementation of NAP-AMR. Behavior change is a strategic objective of the global action plan on AMR and countries’ NAP-AMR, but many countries lack the support needed to achieve this objective. This gap needs more attention, including practical skills-building in applying behavioral interventions. Also, wider base of support for behavioral and other interventions can be garnered by strategically framing AMR as a value-add to existing initiatives rather than as a stand-alone competing priority [33]. Because of their potentially diverse and representative nature, MSC-AMR bodies and their TWGs can play valuable roles in identifying opportunities and facilitating integration or mainstreaming of AMR into existing programs and activities such as HIV/AIDS, tuberculosis, and malaria, safe delivery and neonatal care, and food safety and quality. Leveraging existing One Health platforms or linking to broader plans such as universal health coverage, SDG, and quality improvement can also be instrumental [9]. This can help raise AMR awareness and contribute to the enabling environment needed to facilitate effective MSC-AMR efforts to implement national action plans more comprehensively.

### Lessons learned

Through the WHO’s IHR and GHSA’s ongoing efforts, AMR is increasingly being addressed as a national, regional, and global health security threat. Although the WHO benchmark actions are not intended as an exhaustive list of recommendations, our multi-country experience shows that the tool has filled a key gap in implementation guidance. The benchmark actions have served as an effective and standardized yardstick for measuring countries’ capacity for effective MSC-AMR, allowing countries to move progressively towards the next JEE capacity level.

The existence of a NAP-AMR and MSC-AMR body is necessary but insufficient to address AMR containment [13]. NAPs-AMR must be implemented through a functional MSC body that is supported by effective TWGs that focus on specific technical objective areas such as IPC, AMS, and surveillance. MTaPS’ experience shows some of the critical steps and processes needed for a functional MSC-AMR body, including having and adhering to effective TORs, establishing or strengthening specific TWGs, holding regular meetings with adequate sectoral representation, sharing meeting notes, identifying and facilitating packages of prioritized feasible interventions, and ensuring follow up on actions items. These seemingly simple interventions underpin efforts to strengthen leadership, policy, governance, and capacity building, which are at the cornerstone of MTaPS’ technical approach for strengthening MSC-AMR. Another important consideration for the functionality and sustainability of MSC-AMR bodies is the necessary supporting policies. For some countries, MSC bodies were created through collaboration between government ministries or departments without adequate attention to policies to support their financing and human resources. Additional support is required to formulate needed policies and concomitant resources to address weaknesses in how MSC bodies were created.

Coordinating across and within sectors and creating ownership and collaboration through mutual trust require time and dedicated resources [9]. This presents an even bigger challenge when ministries reorganize or undergo reforms that lead to changes in focal points and membership of MSC bodies. This can delay decision-making and activity progress. When this happens, our mitigation strategy has been to orient new members or ministry personnel, including, where possible, the focal point person being reassigned. More broadly, this challenge underscores the importance of a NAP-AMR with a clearly articulated and applied operational plan and monitoring framework. A systems-based and institutionalized approach to NAP-AMR operationalization guards against shifting priorities and personnel and ensures the continuity of the plan, even when there are big political or administrative changes.

The program has also found that working with stakeholders, including those across and within different levels of ministries, academia, and professional associations, to create strategies, plans, guidelines, and curricula, is an efficient catalyst for sharing knowledge and information to reach targeted players in AMR containment [9]. However, getting to that point of meaningful collaboration entails a series of open discussions to overcome political motivations that may inhibit engagement, increase understanding of the multisectoral context of AMR, and build mutual trust [32, 33]. Results from rapid situational analyses during our scoping visits along with subsequent ongoing discussions formed the basis for building consensus and trust, and identifying, designing and implementing MTaPS-supported interventions. This type of momentum has a better chance of being sustained when committed champions, including both opinion leaders and technical personnel, coalesce and drive country-led actions in a spirit of multisectoral and multidisciplinary collaboration [33]. Furthermore, such coalitions gain further motivation and expansion as results from initial efforts incentivize additional actions that contribute to sustainability.

### Limitations

The paper reports work in progress, and the MSC-AMR bodies have not yet had time to demonstrate a substantial number of outcome-oriented actions. As such, progress, challenges, and lessons learned will continue to evolve during the program. Furthermore, the paper’s scope is restricted to the work of the MTaPS program with the work of other partners included to a limited extent. It therefore does not comprehensively address the MSC-AMR landscape related to support from other partners. In addition, the GHSA mandate of the MTaPS program is limited to MSC-AMR, and IPC and AMS in human health, and the scope of work did not allow this paper to cover the area of AMR surveillance nor adequately incorporate the perspectives of the larger national health security mechanisms.

## Conclusion

MSC is a core policy of the One Health approach aimed at global AMR containment. Given the multiple drivers of AMR, including those rooted in the health, food, animal, and environmental sectors, containing AMR calls for a coordinated response from stakeholders across multiple sectors. How to institutionalize MSC across sectors and between departments that normally work in silos is an ongoing challenge. Developing a NAP-AMR is a substantial step and signifies good political commitment toward AMR containment. However, actually operationalizing it to achieve AMR containment and contribute to GHSA, IHR, universal health coverage, and SDGs will require an adequately resourced and sustained multisectoral approach that is backed by enabling policies, guidelines, and operating procedures. As our findings in this paper show, the JEE tool and WHO benchmarks were highly useful guides to achieving key milestones in AMR containment—finalization of NAPs-AMR and tools to guide their implementation; strengthening the leadership, governance, and oversight capabilities of multisectoral governance structures, including establishment or revitalization of IPC and AMS TWGs; and coordinating AMR activities within and across sectors. This paper therefore provides some practical ideas for countries and their international partners and demonstrates the feasibility of implementing the WHO benchmarks recommended actions to strengthen multisectoral coordination on AMR.

## Data Availability

Not applicable.

## Abbreviations

AMR: Antimicrobial resistance
AMS: Antimicrobial stewardship
COVID-19: Coronavirus disease 2019
CDC: Centers for Disease Control and Prevention
DRC: Democratic Republic of Congo
FAO: Food and Agriculture Organization
GCMN-RAM: Groupe de Coordination Multisectorielle National-Résistance aux Antimicrobiens
GHSA: Global Health Security Agenda
IACG: Interagency Coordination Group
IHR: International Health Regulations
IPC: Infection prevention and control
JEE: World Health Organization’s Joint External Evaluation tool
MOH: Ministry of Health
MPTF: Tripartite Antimicrobial Resistance Multi-Partner Trust Fund
MSC: Multisectoral coordination
MSC-AMR: Multisectoral coordination on antimicrobial resistance
MTaPS: Medicines, Technologies, and Pharmaceutical Services program
NAMRAC: National Antimicrobial Resistance Advisory Committee
NAMRsC: National Antimicrobial Resistance Sub-Committee
NAP-AMR: National action plan on antimicrobial resistance
OIE: World Organisation for Animal Health
SDGs: Sustainable Development Goals
TOR: Terms of reference
TrACSS: Tripartite AMR Country Self-Assessment Survey
TWG: Technical working group
USAID: United States Agency for International Development
WHO: World Health Organization
